# A Shameful Demand by a Hospital Board

**Published:** 1875-02

**Authors:** 


### *A Shameful Demand by a Hospital Board.*

Any one who is the least informed in the current literature of gynæcology, need not be informed of the fact that Dr. Marion Sims has been the founder of that specialty on this continent; nor that his influence and power as a practical teacher has been widely felt throughout the medical world. His knowledge and skill has moulded opinion in this country and Europe. He it was who founded the Woman's Hospital in New York. To his clinics, many men in the profession flocked, eager to hear his words of wisdom, and behold his great skill as an operator. How silly! how unreasonable and unjust, then, that the number of his spectators should, by authority of the Hospital Board, be limited to the small number of fifteen! Dr. Sims, feeling the injustice of so daft a ruling, promptly tendered his resignation, as would any man of spirit have done, under like circumstances. It is to be regretted, for his power as a teacher is thus greatly abridged. Let it be hoped that the medical press throughout the world will sustain him in the course he has chosen. We hope that no man in New York will be induced, for any consideration, to accept the position he has vacated while this absurd ruling continues in force. Better would it be for the Board to widen his useful sphere, than to undertake to restrict it in such an unreasonable manner. To no man living are the women of the world as much indebted as to Marion Sims.

